# Composition of the Vaginal Microbiota in Women of Reproductive Age – Sensitive and Specific Molecular Diagnosis of Bacterial Vaginosis Is Possible?

**DOI:** 10.1371/journal.pone.0060670

**Published:** 2013-04-09

**Authors:** Elena Shipitsyna, Annika Roos, Raluca Datcu, Anders Hallén, Hans Fredlund, Jørgen S. Jensen, Lars Engstrand, Magnus Unemo

**Affiliations:** 1 Laboratory of Microbiology, D. O. Ott Research Institute of Obstetrics and Gynecology, Saint Petersburg, Russia; 2 Department of Microbiology, Tumor and Cell Biology, Karolinska Institutet, Solna, Sweden; 3 Department of Microbiological Surveillance and Research, Statens Serum Institut, Copenhagen, Denmark; 4 Department of Dermatology and Venereology, Uppsala University Hospital, Uppsala, Sweden; 5 World Health Organization Collaborating Centre for Gonorrhoea and other Sexually Transmitted Infections, Swedish Reference Laboratory for Pathogenic Neisseria, Örebro University Hospital, Örebro, Sweden; Ghent University, Belgium

## Abstract

**Background and Objective:**

Bacterial vaginosis (BV) is the most common vaginal disorder, characterized by depletion of the normal lactobacillus-dominant microbiota and overgrowth of commensal anaerobic bacteria. This study aimed to investigate the composition of the vaginal microbiota in women of reproductive age (healthy women and women with BV), with the view of developing molecular criteria for BV diagnosis.

**Materials and Methods:**

Vaginal samples from 163 women (79 control, 73 BV and 11 intermediate (Lactobacillary grade II flora) cases) were analyzed using 454 pyrosequencing of the hypervariable regions V3–V4 of the 16S rRNA gene and 16 quantitative bacterial species/genus-specific real-time PCR assays. Sensitivities and specificities of potential BV markers were computed using the Amsel criteria as reference standard for BV. The use of quantitative thresholds for prediction of BV, determined for both relative abundance measured with 454 pyrosequencing and bacterial load measured with qPCR, was evaluated.

**Results:**

Relative to the healthy women, the BV patients had in their vaginal microbiota significantly higher prevalence, loads and relative abundances of the majority of BV associated bacteria. However, only *Gardnerella vaginalis*, *Atopobium vaginae*, *Eggerthella*, *Prevotella*, BVAB2 and *Megasphaera* type 1 detected at or above optimal thresholds were highly predictable for BV, with the best diagnostic accuracy shown for *A. vaginae*. The depletion of *Lactobacillus* species combined with the presence of either *G. vaginalis* or *A. vaginae* at diagnostic levels was a highly accurate BV predictor.

**Conclusions:**

Quantitative determination of the presence of *G. vaginalis*, *A. vaginae*, *Eggerthella*, *Prevotella*, BVAB2 and *Megasphaera* type 1 as well as the depletion of *Lactobacillus* was highly accurate for BV diagnosis. Measurements of abundance of normal and BV microbiota relative to total bacteria in vaginal fluid may provide more accurate BV diagnosis, and be used for test-of-cure, rather than qualitative detection or absolute counts of BV related microorganisms.

## Introduction

Bacterial vaginosis (BV) is the most common vaginal disorder in women of reproductive age [1], commonly manifested as abnormal vaginal discharge. BV is associated with adverse health outcomes in both pregnant and non-pregnant women, which include preterm delivery [2], amniotic fluid infections [3], chorioamnionitis [4], pelvic inflammatory disease (PID) [5], [6], [7], [8], cervicitis [9], [10] and increased susceptibility to infection with various pathogens, such as *Neisseria gonorrhoeae*, *Chlamydia trachomatis*, *Trichomonas vaginalis*, HSV-2 and HIV [11], [12], [13], [14].

No single etiologic agent is known to be the cause of BV, and the syndrome is considered an ecological disorder of the vaginal microbiota. It is characterized by depletion of the normal lactobacillus-dominant microbiota, accompanied by a shift towards higher bacterial diversity and intense overgrowth of commensal anaerobic bacteria (100–1000-fold above normal) [15]. Traditional cultivation has identified numerous BV associated bacteria, including *Gardnerella vaginalis*, *Prevotella* spp., *Porphyromonas* spp., *Bacteroides* spp., *Peptostreptococcus* spp., *Mobiluncus* spp. and *Mycoplasma hominis* (reviewed in [16]). However, the true extent of the microbial diversity in BV has been indicated only with the advent of cultivation-independent molecular-based approaches, such as taxon-directed PCR, fluorescence in situ hybridization (FISH) and most widely used broad-range bacterial 16S rDNA PCR, with subsequent separation of 16S rDNA PCR products using denaturing gradient gel electrophoresis (DGGE) of amplicons, terminal restriction fragment length polymorphism (T-RFLP) analysis, cloning of PCR products with direct sequencing or high throughput pyrosequencing [17], [18], [19], [20], [21], [22], [23]. With the use of molecular methods, several novel bacteria (of which some remain uncultivated), including *Atopobium vaginae*, *Megasphaera* spp., *Leptotrichia* spp., *Dialister* spp. and the three bacterial vaginosis associated bacteria BVAB1, BVAB2, BVAB3 that belong to the order *Clostridiales,* have been shown to be associated with BV [18], [24], [25].

In clinical practice, BV is typically diagnosed using the Amsel criteria, which include the presence of at least three of the following four findings: homogeneous vaginal discharge, vaginal pH greater than 4.5, “fishy” odor on addition of potassium hydroxide to vaginal fluid (positive “whiff test”), and presence of clue cells in microscopy of wet preparation [26]. BV may also be diagnosed using a score, the Nugent criteria, assessing the proportion of lactobacilli in relation to BV associated bacterial morphotypes (*Gardnerella/Bacteroides* and *Mobiluncus*) in Gram stained preparations of vaginal fluid or mucus. The resulting scale ranges from normal (0–3) through intermediate (score 4–6) to BV (score 7–10) [27]. Other Gram staining diagnostic criteria, based on the estimation of the ratios of observed morphotypes rather than their exact numbers, have also been described [28], [29]. As well, a classification based on the relative quantities of lactobacillary morphotypes in wet mount microscopy (Lactobacillary grade) has been proposed for detection of abnormal vaginal flora including partial or full BV [30]. The Amsel criteria, wet mount microscopy and the Nugent score on Gram stained preparations are the three most widely used diagnostic methods, which are often applied in research studies; however, these methods remain underutilized in routine diagnosis of BV [31]. Insufficient appropriate use of the Amsel criteria and wet mount microscopy in clinical practice is often due to clinicians’ lack of or restricted skill in using microscopy of wet mount preparation to detect typical BV flora and clue cells. The adoption of the Nugent score by clinical laboratories is limited by its complexity and subjectivity, and this method does not permit the identification of several bacterial morphotypes associated with BV [32]. Furthermore, the proportion of samples assigned to the Nugent category of intermediate vaginal microbiota may exceed 20% [33], [34], and it remains debated how to handle these results [35]. Given the substantial adverse effect that BV may have on women’s reproductive health, there is a need for rapid, accurate and objective methods for diagnostics including test-of-cure.

The use of taxon-directed PCR assays for the diagnosis of BV has been explored in a few studies, and several bacterial species have been shown to be useful as BV predictors [18], [19], [31], [33], [36], [37], [38]. Different BV associated bacteria have divergent predictive values for the diagnosis of BV, and combined detection of several of these bacteria might increase these values [18], [19], [31]. Improvement of diagnostic accuracy might also be gained using quantitative thresholds of BV related bacteria [31]. However, although several molecular approaches appear promising as diagnostic tools, a highly sensitive and specific method for molecular diagnosis of BV remain lacking.

The aim of the present study was to investigate in detail the composition of the vaginal microbiota in women of reproductive age (healthy women and women with BV), using massive parallel 454 pyrosequencing and 16 quantitative bacterial species/genus-specific real-time PCR assays (qPCR), with the view of developing molecular criteria for the diagnosis of BV.

## Materials and Methods

### Patients and Specimen Collection

From October 2009 to June 2010, 163 unselected women attending the Department of Dermatovenereology, Uppsala University Hospital, Sweden for regular check-ups (n = 90) or due to vaginal complaints (discharge, itching, burning, dysuria) (n = 73) that agreed to participate and full-filled the inclusion criteria were enrolled in the study. Accordingly, all women were non-pregnant, of reproductive age (range, 15–54 years; mean, 26 years; median, 24 years), had not been using antimicrobials (oral or topical) within the previous 4 weeks, and had not been using an intrauterine device or contraceptives delivered directly to the vaginal mucosa. All women received treatment for BV if the condition was diagnosed clinically.

After receiving written informed consent from each included woman, the woman was interviewed and two samples were collected from the vaginal fornix using 10 µl plastic loops. One sample was used for a wet mount preparation; the other sample, to be analyzed by 454 pyrosequencing and qPCR, was placed in 0.7 ml of sterile phosphate-buffered saline, supplemented with 10% glycerol, vortexed and then immediately frozen at −70°C.

### Ethical Approval

The study protocol was approved by the Ethics Committee, Uppsala University, Sweden (Dnr 2012/249).

### Diagnosis of BV

For defining BV, the Amsel criteria [26] were used. A subject was categorized as healthy if less than three of the Amsel criteria were found, and as having BV if three or four of the Amsel criteria were present. Furthermore, a case was considered intermediate if, irrespective of the clinical characteristics and Amsel criteria, mixed flora consisting of cocci and rods, with rare clue cells, was observed in wet mount microscopy, which corresponds to the partial BV category in Lactobacillary grade II flora in the Lactobacillary grade classification [35] and should not be confused with the intermediate category in the Nugent score.

### DNA Extraction

DNA was extracted from 100 µl of each sample using DNeasy blood and tissue kit (Qiagen, Hilden, Germany), including Proteinase K incubation, according to the manufacturer’s instructions. Final elution was made with 100 µl (2×50 µl) of elution buffer. All DNA extracts were stored at −20°C prior to sequencing and qPCR.

### 454 Pyrosequencing, Sequence Processing and Classification

For each sample, three 50 µl PCR mixes were prepared, containing 1×PCR buffer, 200 µM dNTP PurePeak DNA polymerization Mix (all from Pierce Nucleic Acid Technologies, Milwaukee, USA), 25 µM of each primer, 0.5 U Phusion F-530L enzyme (Finnzyme, Massachusetts, USA) and 1 µl of template DNA. The primer pairs used to amplify the hypervariable 16 rDNA regions V3–V4 were 341f (5′CCTACGGGNGGCWGCAG) with adaptor B and 805r (5′GACTACHVGGGTATCTAATCC) with adaptor A, and a sample specific sequence tag of 7 nucleotides for the Genome Sequencer (GS) FLX Titanium (Roche, Arizona, USA) run. A PCR negative template control was also used for each primer pair. The PCR conditions were 95°C for 5 minutes, followed by 25 cycles of 95°C for 40 sec, 58°C for 40 sec, and 72°C for 1 min, and finally an extension step at 72°C for 7 min. The samples and the negative template control were then analyzed on an agarose gel (1% w/v in TBE buffer) for quality control. The three PCR reactions were pooled and 45 µl of each pooled PCR reaction was purified using Agencourt AMPure beads (Beckman Coulter, California, USA) according to the manufacturer’s instructions with final elution in 15 µl of 1×TE buffer. DNA concentration was measured using a Qubit kit (Invitrogen, California, USA). The samples were adjusted to a concentration of 3 ng/µl, and 5 µl of each sample were added into a mix for 454 pyrosequencing. The 454 pyrosequencing was performed with a standard amplicon kit and run on a 454-FLX GS-l00, using the GS FLX Titanium protocol (Roche, Arizona, USA) [39].

Sequences were excluded when there was no perfect match with primer or barcode, if the length was less than 500 nucleotides, or if there were ambiguous nucleotides. Non-redundant reads after removal of the primer/barcode sequence were aligned and sorted into operational taxonomic units (OTUs) with a 3% distance threshold. This was performed with tools (Pyrosequencing Pipeline and Complete Linkage Clustering) available at the Ribosomal Database Project (RDP) at http://rdp.cme.msu.edu [40]. The Complete Linkage Clustering was performed using a maximum cluster distance of 50 and step 1.

16S rDNA sequences downloaded from RDP (release 10, update 22) were converted into a local BLAST database. An identification threshold of 95% over at least 180 nucleotides was used for the OTU BLASTing against the database. OTUs with no significant hit were assigned “no_match” and if there were several hits, the OTU was specified as the lowest level of common hits. The OTU hits were sorted into taxonomic level as well as phylum level for further analysis.

### Quantitative Bacterial Species/genus-specific Real-time PCR Assays

Sixteen bacterial species/genera were selected for the detection by qPCR based on their relevance for BV according to previous studies [17], [18], [19], [20], [21], [22], [23], [41]. Five species (*G. vaginalis, A. vaginae, Sneathia sanguinegens, Leptotrichia amnionii, Lactobacillus iners*) were detected using TaqMan based PCR assays; for the remaining 11 species/genera (*Eggerthella*, *Prevotella,* BVAB1, BVAB2, BVAB3, *Finegoldia magna*, *Megasphaera* type 1, *Megasphaera* type 2, BVAB-TM7, *Mobiluncus curtisii* and *M. mulieris*) SYBR Green based PCR assays were used. The oligonucleotide primers targeting the 16S rRNA genes of the bacteria are listed in Table S1 (this study), [19], [42], [43], [44]. Specificities of all newly designed primers and probes were confirmed by BLAST searches in the GenBank (http://www.ncbi.nlm.nih.gov/BLAST/).

Each TaqMan PCR reaction mixture contained 1×PCR Buffer, 5 mM MgCl_2_, 125 µM each of dATP, dCTP and dGTP, 250 µM dUTP, 2 U of Platinum Taq DNA polymerase (all from Invitrogen by Life Technologies), 1 µM each of forward and reverse primers, 75 nM probe and 5 µl of template DNA in a total volume of 50 µl. The thermocycling conditions for all TaqMan based PCR assays were 95°C for 10 min, followed by 50 cycles of 95°C for 15 seconds and 60°C for 1 min, with fluorescence read at 60°C.

Each SYBR Green PCR reaction mixture contained 1×PCR Buffer, 1.5–3.5 mM MgCl_2_ (MgCl_2_ concentration for each assay is listed in Table S1), 2 U of Platinum Taq DNA polymerase, 125 µM each of dATP, dCTP and dGTP, 250 µM dUTP (all from Invitrogen by Life Technologies), SYBR Green (Life technologies) 1∶5000 dilution, 0.4 µM each of forward and reverse primers and 5 µl of template DNA in a total volume of 50 µl. The thermocycling profile for all SYBR Green based PCR assays, with the exception of the PCR assays for *M. curtisii* and *M. mulieris*, was: 95°C for 10 min; 10 cycles of 95°C for 15 seconds, 65°C to 55°C touchdown with a 1°C decrement per cycle for 30 seconds, 72°C for 32 seconds; 30 cycles of 95°C for 15 seconds, 55°C for 30 seconds, 72°C for 32 seconds; and finally 72°C for 7 min, followed by a melting curve analysis. For the *M. curtisii* and *M. mulieris* PCR assays, the thermocycling conditions were identical to those for the other SYBR Green assays except that during the touchdown phase the temperature decreased from 70°C to 60°C, and further elongation was performed at 60°C. All assays were performed using ABI 7500 Sequence Detection Systems with 96 well conventional blocks (ABI/Life Technologies).

For quantification of the bacterial species/genera, genomic DNA from cultivable species was extracted and quantified using a Qubit Kit (Invitrogen). For BVAB 1, 2, 3, BVAB-TM7, *Megasphaera* type 1 and 2, and *Eggerthella*-like uncultured bacterium, the PCR product from a positive clinical specimen was used as positive control after gel-purification and DNA sequencing for verification. Standard curves were generated using 10-fold dilutions from 1 genome equivalent (geq)/µl to 10^7 ^geq/µl in TE buffer (10 mM Tris HCl, pH 8.0 with 1 mM EDTA containing 1 µg/ml of calf thymus DNA (Sigma-Aldrich)). Assay results were expressed as genome/gene copy numbers per 1 µl of DNA extract. Negative controls were included in each PCR run.

### Data Analyses

The pyrosequencing results on taxonomy and phylum levels were visualized with regard to relative abundance as a heat map in MultiExperiment Viewer 4.0. (available at http://www.tm4.org/mev/). The analysis was based on the Pearson correlation using absolute distances.

Descriptive statistics used for summarizing quantitative 454 pyrosequencing and species/genus-specific PCR results included mean and median values. For testing differences between the groups of patients, chi-square statistics were computed for categorical variables (presence of bacterial species/genera), and the Mann-Whitney U test was performed for continuous variables (relative abundances and DNA concentrations of bacteria). All tests for significance were two-sided, and statistically significant differences were assumed when P<0.05.

Agreement between the presence of an individual bacterial species/genus determined by 454 pyrosequencing and by qPCR was measured using Cohen’s kappa, with magnitude of kappa categorized as indicating poor (<0.40), fair to good (0.40 to 0.75) or excellent (>0.75) agreement [45].

Diagnostic characteristics of potential BV markers were computed using the Amsel criteria as reference standard for BV. For qualitative detection of bacterial species/genera, sensitivities and specificities were computed. For quantitative data (relative abundance of bacteria measured with 454 pyrosequencing and bacterial load measured with qPCR), receiver operating characteristic (ROC) curves were plotted, and sensitivities and specificities were determined at optimal thresholds. As well, areas under the curve (AUCs) were defined to characterize diagnostic accuracy of quantitative BV markers. Diagnostic accuracy was categorized as failed (ROC AUC ≤0.6), poor (0.6< ROC AUC ≤0.7), fair (0.7< ROC AUC ≤0.8), good (0.8< ROC AUC ≤0.9) or excellent (0.9< ROC AUC ≤1.0). Statistical analyses were performed using the statistical software packages R (http://CRAN.R-project.org/package=vegan), StatsDirect version 2.7.2 (StatsDirect) and SPSS version 17.0 (SPSS).

## Results

### The Composition of the Vaginal Microbiota by 454 Pyrosequencing

**Figure 1:**
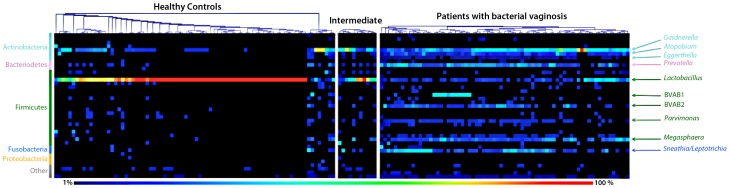
Heat map based on taxon visualized with MultiExperiment Viewer. The figure shows clustering of the samples based on the Pearson correlation using absolute distances and complete linkage clustering with a cut-off level set to 1%. The individuals are shown on the x-axis and the different taxons on the y-axis. The relative abundance of each taxon is correlated to the color of the dot, where black is 1% and red is 100%. The most distinct taxons are shown to the right, colored according to the different phyla to the left.

**Figure 2:**
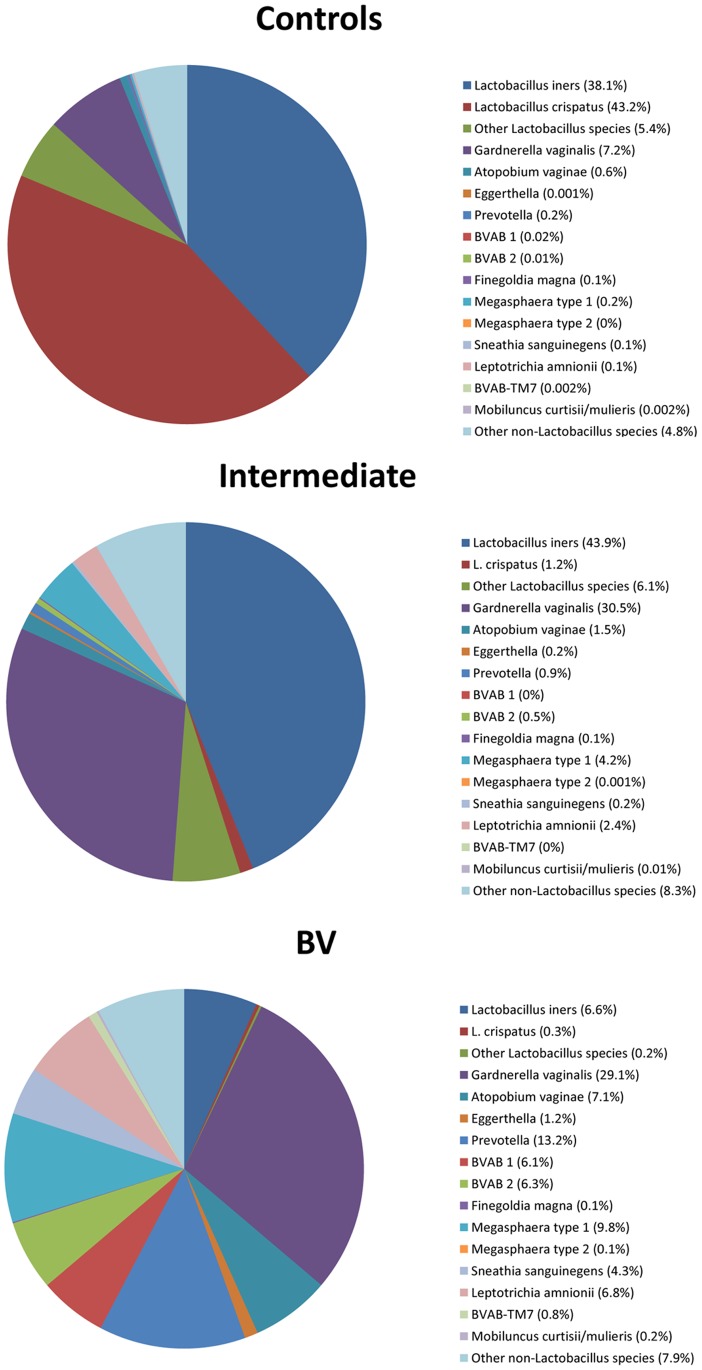
Relative abundances of bacterial species/genera by 454 pyrosequencing. The figure presents the average relative abundances of 16 bacterial species/genera by 454 pyrosequencing in the vaginal samples from healthy control women (n = 79), intermediate cases (n = 11) and BV patients (n = 73). The healthy control women had vaginal microbiota dominated by *Lactobacillus* species, accounting for nearly 90% of all sequences, with *L. crispatus* and *L. iners* highly prevailing over other lactobacilli. In the intermediate cases, *L. iners* and *G. vaginalis* were dominating over other species, accounting together for nearly 75% of all sequences. The BV patients possessed a diverse array of bacteria, with the most abundant species being *G. vaginalis*, *Prevotella*, *Megasphaera* type 1, *A. vaginae*, *L. amnionii*, *L. iners*, BVAB1, BVAB2, *S. sanguinegens* and *Eggerthella*.

Of the 163 women enrolled in the study, 79 were assigned as healthy control subjects, 73 were diagnosed with BV and 11 were categorized as intermediate cases. The sequencing results, visualized as a heat map with a clustering of the samples based on the Pearson correlation and with a relative abundance cut-off set to 1%, showed extensive differences between these groups of subjects (Figure 1). Most of the healthy control women had a vaginal microbiota totally dominated by *Lactobacillus,* with BV associated taxons hardly detectable. The dominating taxons in the intermediate cases were *Lactobacillus* and *Gardnerella.* A few of the healthy control women (the phylogenetic cluster in the right end of the block of healthy women) appeared to have a microbiota changing towards an intermediate microflora. The BV patients formed a heterogeneous group, with high proportions of *Gardnerella*, *Atopobium*, *Eggerthella, Prevotella,* BVAB1, BVAB2, *Lactobacillus, Megasphaera* and *Sneathia*/*Leptotrichia*. There were dramatic differences between the three groups of subjects in the relative abundances of vaginal bacterial species/genera (Figure 2, Table S2). The healthy control women had vaginal microbiota dominated by *Lactobacillus* species, accounting on average for nearly 90% of all sequences, with *L. crispatus* and *L. iners* highly prevailing over other lactobacilli. In the intermediate cases, *L. iners* and *G. vaginalis* were dominating over other species, accounting together for nearly 75% of all sequences. The BV patients possessed a diverse array of bacteria, with the most abundant species/genera being *G. vaginalis* (29% of all sequences), *Prevotella* (13%), *Megasphaera* type 1 (10%), *A. vaginae* (7%), *L. amnionii* (7%), *L. iners* (7%), BVAB1 (6%), BVAB2 (6%), *S. sanguinegens* (4%) and *Eggerthella* (1%).

### Qualitative and quantitative analyses of vaginal bacteria by species/genus-specific real-time PCR

**Figure 3:**
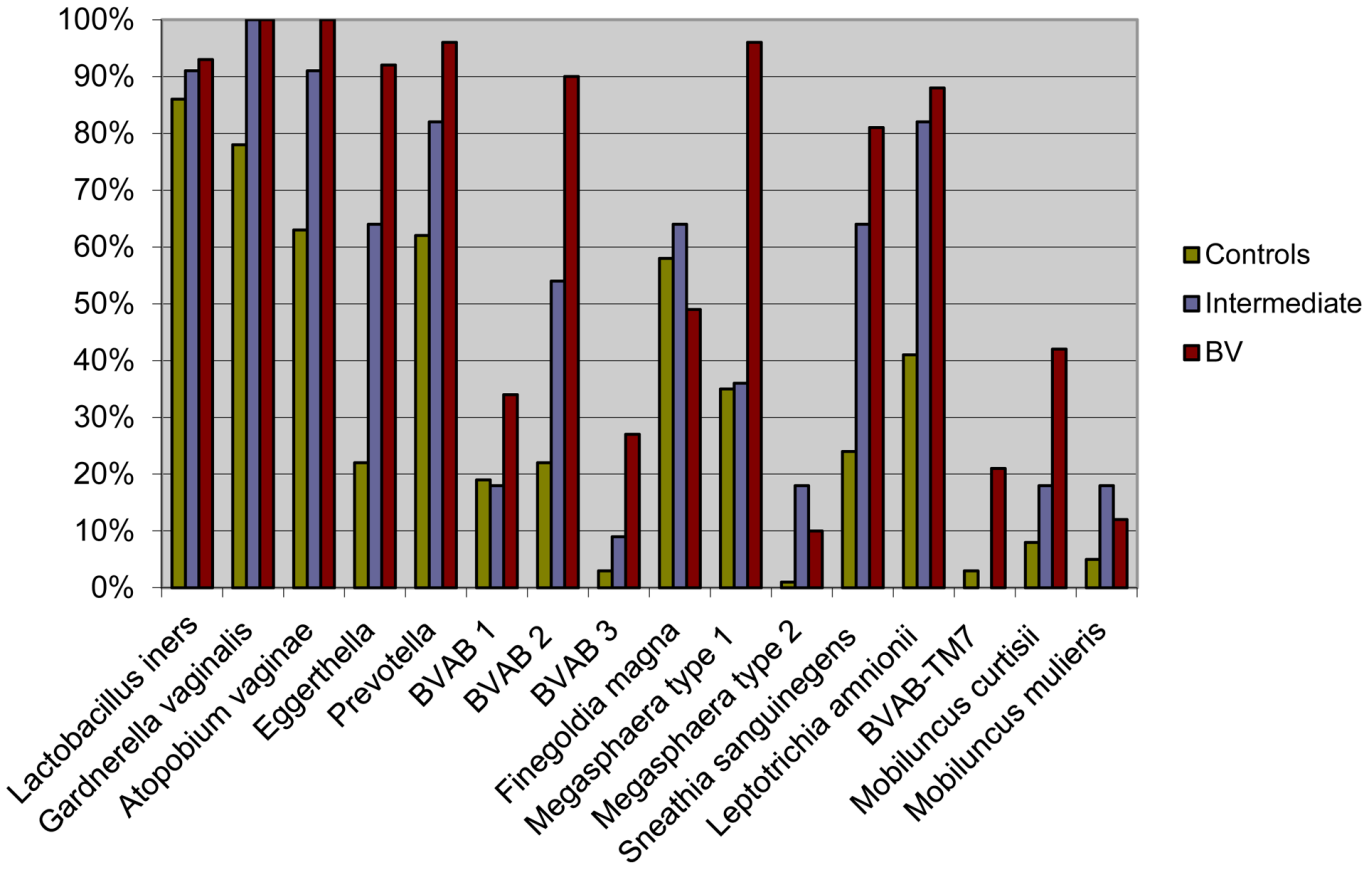
Prevalence of vaginal bacterial species/genera by real-time PCR. The qualitative detection rates of 16 bacteria by species/genus-specific PCR assays in healthy control women (n = 79), intermediate cases (n = 11) and BV patients (n = 73) are presented. *L. iners* was the most frequently detected bacterium in the healthy control women (86%), followed by *G. vaginalis* (78%), *A. vaginae* (63%), *Prevotella* (62%), and *F. magna* (58%). In the intermediate subjects, the most common species/genera were *G. vaginalis* (100%), *L. iners* (91%), *A. vaginae* (91%), *Prevotella* (82%) and *L. amnionii* (82%). *G. vaginalis* and *A. vaginae* were both found in 100% of the subjects with BV. Other highly prevalent bacteria in the BV patients were *Prevotella* (96%), *Megasphaera* type 1 (96%), *L. iners* (93%), *Eggerthella* (93%), BVAB2 (90%), *L. amnionii* (88%) and *S. sanguinegens* (81%).

The qualitative detection rates of the 16 bacteria by species/genus-specific PCR assays are presented in Figure 3. *L. iners* was the most frequently detected bacterium in the healthy control women (86%), followed by *G. vaginalis* (78%), *A. vaginae* (63%), *Prevotella* spp. (62%), and *F. magna* (58%). In the intermediate subjects, the most common species/genera were *G. vaginalis* (100%), *L. iners* (91%), *A. vaginae* (91%), *Prevotella* spp. (82%) and *L. amnionii* (82%). *G. vaginalis* and *A. vaginae* were both found in 100% of the subjects with BV. Other highly prevalent bacteria in the BV patients were *Prevotella* spp. (96%), *Megasphaera* type 1 (96%), *L. iners* (93%), *Eggerthella* spp. (93%), BVAB2 (90%), *L. amnionii* (88%) and *S. sanguinegens* (81%).

The three groups of subjects did not differ significantly in the qualitative detection rates of *L. iners*, *F. magna* and *M. mulieris*. For the remaining bacteria, the frequency of detection was significantly higher in the BV patients versus the control subjects (Figure 3, Table S3).

**Figure 4:**
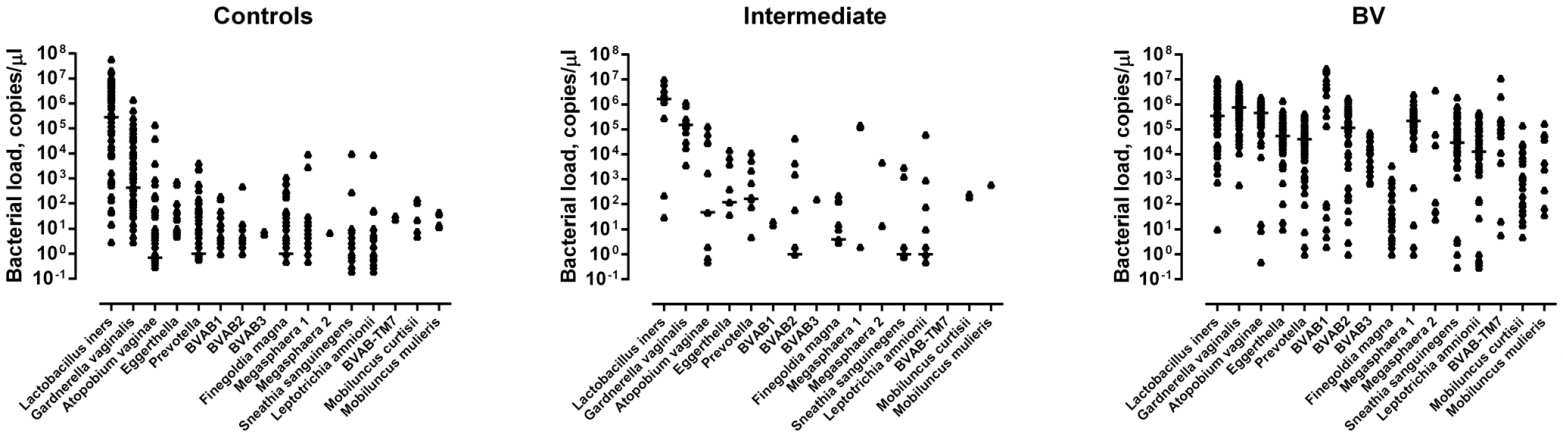
Genome/gene copy numbers determined by specific quantitative PCR of bacterial species/genera in vaginal samples by species/genus-specific qPCR. The scatter plots present DNA concentrations of the 16 bacteria by qPCR in the samples from healthy control women (n = 79), intermediate cases (n = 11) and BV patients (n = 73). Bars represent median concentrations. *L. iners* had the highest median concentration in the samples from both healthy (2.8×10^5^ copies/µl) and intermediate (1.7×10^6^ copies/µl) subjects. In the intermediate subjects, also *G. vaginalis* was present in high loads (median concentration 1.5×10^5^ copies/µl). In the BV subjects, the highest median concentrations were displayed by *G. vaginalis* and *A. vaginae* (7.6×10^5^ and 4.6×10^5^ copies/µl, respectively), followed by *L. iners, Megasphaera* type 1 and BVAB2 (3.5×10^5^, 2.2×10^5^ and 1.2×10^5^ copies/µl, respectively).

The quantitative PCR results are summarized in scatter plots presenting concentrations of specific DNA of the 16 bacterial species/genera in samples obtained from the healthy control women, intermediate cases and BV patients (Figure 4). *L. iners* had the highest median concentration of any assayed species/genus in samples from both healthy control women (2.8×10^5^ copies/µl) and intermediate cases (1.7×10^6^ copies/µl). In the intermediate cases, also *G. vaginalis* was present in high loads (median concentration 1.5×10^5^ copies/µl). In the BV patients, the highest median concentrations were displayed by *G. vaginalis* and *A. vaginae* (7.6×10^5^ and 4.6×10^5^ copies/µl, respectively), followed by *L. iners, Megasphaera* type 1 and BVAB2 (3.5×10^5^, 2.2×10^5^ and 1.2×10^5^ copies/µl, respectively).

There was no significant difference between the three groups of subjects in regard to loads of *L. iners*, *F. magna* and *M. mulieris*. For the remaining bacteria, the concentrations were significantly higher in the BV patients as compared to the healthy control women (Figure 4, Table S3).

Measurements of agreement between the presence of an individual species/genus determined by 454 pyrosequencing versus real-time PCR are presented in Table S4. For the majority of species/genera, the kappa values indicated fair to good agreement. Most cases of disagreement were samples negative by 454 pyrosequencing but positive by real-time PCR, which can be ascribed to the higher sensitivity of the PCR assays. In the vast majority of the discrepant cases, the quantity/abundance of bacteria was very low, i.e., substantially below the optimal diagnostic threshold values determined using ROC curve analysis.

### Diagnostic Performance of Potential BV Markers

Using the Amsel criteria for defining BV, sensitivity and specificity of qualitative detection of different bacterial species/genera for BV diagnosis were computed. For quantitative data, sensitivity and specificity at the optimal diagnostic thresholds were determined, and diagnostic accuracy was defined (Table S5). Among the non-*Lactobacillus* species, qualitative PCR detection was highly sensitive for BV prediction when targeting *G. vaginalis, A. vaginae* and *Prevotella* spp. (100%, 100% and 96%, respectively), but these species/genera were also detected in the majority of healthy control women, resulting in poor specificity (22%, 37% and 38%, respectively). In contrast, *Megasphaera* type 2, BVAB3, BVAB-TM7, *M. mulieris* and *M. curtisii* were highly specific for BV (99%, 97%, 97%, 95% and 92%, respectively); however, as these bacteria were present in a small proportion of the BV patients, the sensitivity of BV prediction was very low (10%, 27%, 23%, 12% and 43%, respectively). The best combinations of sensitivity and specificity were shown for the detection of three bacterial species/genera, namely, *Megasphaera* type 1, *Eggerthella*, BVAB2 (sensitivity 96%, 93% and 90%, respectively, and specificity 65%, 78% and 78%, respectively). The detection of *S. sanguinegens* and *L. amnionii* predicted BV with a sensitivity of 88% and 81%, respectively, and a specificity of 59% and 76%, respectively. *F. magna* was detected in 49% of the subjects with BV (49% sensitivity) and in 58% of the subjects without BV (42% specificity), thus showing no discriminatory ability for BV.

The use of optimal quantitative diagnostic threshold values, for both bacterial loads measured with qPCR and relative abundances measured with 454 pyrosequencing, substantially improved the discriminatory abilities of several bacterial BV markers (Table S5). There was a substantial increase in the specificity of the detection of the bacteria highly prevalent in BV, namely, *G. vaginalis*, *A. vaginae*, *Eggerthella* spp., *Prevotella* spp., BVAB2 and *Megasphaera* type 1, with only a slight decrease in the sensitivity. For qPCR assays, the highest diagnostic accuracy (ROC AUC 0.99) was displayed by the detection of *A. vaginae*. At the optimal diagnostic threshold (≥7930 copies/µl), the test predicted BV with 96% sensitivity and 97% specificity. Excellent diagnostic accuracy was also shown for the *G. vaginalis* qPCR assay (ROC AUC 0.96; sensitivity 88% and specificity 95% at ≥120310 copies/µl), *Eggerthella* assay (ROC AUC 0.96; sensitivity 90% and specificity 97% at ≥105 copies/µl), *Megasphaera* type 1 assay (ROC AUC 0.96; sensitivity 88% and specificity 100% at ≥17405 copies/µl), *Prevotella* assay (ROC AUC 0.94; sensitivity 89% and specificity 94% at ≥560 copies/µl) and BVAB2 assay (ROC AUC 0.93; sensitivity 86% and specificity 99% at ≥20 copies/µl). The qPCR detection of *S. sanguinegens* and *L. amnionii* possessed somewhat lower diagnostic accuracy (ROC AUC 0.86 and 0.89, respectively). The qPCR assays for the other bacteria showed only poor to failed accuracy.

When ROC curves were plotted for the relative abundances of the bacteria, excellent diagnostic accuracy among the non-*Lactobacillus* species was identified for *A. vaginae* (ROC AUC 0.97; sensitivity 96% and specificity 97% at a proportion of ≥0.7%), *Prevotella* (ROC AUC 0.97; sensitivity 92% and specificity 94% at ≥0.8%), *Eggerthella* (ROC AUC 0.92; sensitivity 85% and specificity 100% at ≥0.1%) and *Megasphaera* type 1 (ROC AUC 0.93; sensitivity 90% and specificity 97% at ≥0.4% (Table S5). The diagnostic accuracy of the detection of *G. vaginalis*, BVAB2, *S. sanguinegens* and *L. amnionii* was categorized as good (ROC AUC 0.89, 0.88, 0.85 and 0.86, respectively). The quantitative 454 pyrosequencing results for the remaining bacteria showed only poor to failed accuracy.

Diagnostic characteristics of the detection of *Lactobacillus* species, as negative BV predictors, were also analyzed (Table S5). The absence of *L. iners*, as determined by qualitative PCR, predicted BV with only 7% sensitivity (and 86% specificity), and the detection of *L. iners* by qPCR also showed no discriminatory ability for BV (ROC AUC 0.51). The quantitative 454 pyrosequencing detection of *L. iners* had somewhat higher accuracy (ROC AUC 0.65), yet the specificity of the assay at the optimal diagnostic threshold (≤35%) was very low (46%). The absence of *L. crispatus* (only 454 pyrosequencing data were available for *L. crispatus* and *Lactobacillus* spp.) predicted BV with a sensitivity of 66% and a specificity of 82%. With a diagnostic threshold established at a relative abundance of 0.3%, the sensitivity of BV prediction dramatically increased (to 96%), but the specificity decreased to 70%, since a large proportion of the healthy control women (30%) were also either completely lacking *L. crispatus* DNA or containing it at a relative abundance of ≤0.3%. *Lactobacillus* spp. showed no discriminatory ability for BV diagnosis when detected on a qualitative basis, i.e., all control subjects had *Lactobacillus* spp. (specificity 100%), but this was the case also for 96% of the BV patients (sensitivity 4%). However, when a threshold value was established at ≤47%, the sensitivity increased to 100%, with high specificity (94%) retained.

In order to improve the accuracy of BV diagnosis, the detection of the two most prevalent BV associated species, i.e., *G. vaginalis* and *A. vaginae*, and *Lactobacillus* spp., in different combinations, was also evaluated (Table S6). The highest sensitivities and specificities were obtained when the depletion of *Lactobacillus* spp. (relative abundance ≤47%) was combined with the presence of either *G. vaginalis* or *A. vaginae* at diagnostic levels, as determined by qPCR (sensitivity 97%, specificity 96%), and as determined by 454 pyrosequencing (sensitivity 100%, specificity 95%).

## Discussion

Comprehensive knowledge of the composition of the vaginal microbiota in BV patients as compared to healthy women is essential for understanding the etiology of BV and for the development of diagnostic tools, effective treatment and possibly prevention of this disease. The results of our study supported previous studies that BV is associated with dramatic compositional changes in the vaginal microbiota, i.e., the depletion of lactobacilli in conjunction with the massive colonization of the vagina with many diverse bacteria, mainly strict anaerobes [17], [18], [19], [20], [21], [22], [23], [24], [41], [46], [47]. In the present study, it was also shown that, relative to the healthy control women, the BV patients had in their vaginal microbiota significantly higher prevalence, loads and relative abundances of the majority of the BV associated bacteria assayed. However, only six bacterial species/genera, namely, *G. vaginalis*, *A. vaginae*, *Eggerthella, Prevotella,* BVAB2 and *Megasphaera* type 1 were shown to be potentially valuable for BV diagnosis, since they were present in most BV patients, which ensured high diagnostic sensitivity.

In our study, both *G. vaginalis* and *A. vaginae* were detected by real-time PCR in 100% of the BV patients, but these bacteria were also detected in 78% and 63% of subjects without BV, respectively, confirming that the qualitative detection of these species had very low specificity (22% and 37%, respectively). The use of quantitative diagnostic thresholds resulted in a dramatic improvement of the diagnostic characteristics. The qPCR yielded 88% sensitivity and 95% specificity for *G. vaginalis* and 96% sensitivity and 97% specificity for *A. vaginae*. With the use of 454 pyrosequencing thresholds, the sensitivity and specificity of BV detection was 96% and 78%, respectively, for *G. vaginalis*, and 96% and 97%, respectively, for *A. vaginae*. Our data are consistent with the results of Menard et al. [31]. These two species co-occur in the large majority of BV patients, and the possibility of combining the detection of these two species for an accurate diagnosis of BV has been suggested [17], [31], [48].

*Eggerthella* spp. was also highly predictable for BV in our study. The detection of *Eggerthella* spp. even on a qualitative basis possessed relatively good diagnostic characteristics (93% sensitivity and 78% specificity), and the quantitative detection was highly accurate for BV (ROC AUCs 0.96 and 0.92 for qPCR and 454 pyrosequencing, respectively). Fredricks et al. [19] also showed that the detection of this bacterium using qualitative PCR was both sensitive (91%) and specific (86%) for BV diagnosis. Tamrakar et al. [47] reported that the presence of *Eggerthella* was an independent risk factor of BV. In the study of Srinivasan et al. [23], *Eggerthella* was one of the two bacteria (the other was *L. amnionii*) that were positively associated with all four Amsel criteria.

Our data also support previous studies showing that *Prevotella* spp. was a highly prevalent and abundant microorganism in the vagina [20], [21], [44], [49]. With our qPCR, *Prevotella* spp. was detected in 62%, 82% and 96% of the healthy control women, intermediate cases and BV patients, respectively. *Prevotella* was the second, after *G. vaginalis*, most abundant bacterium in the samples from the BV patients, reaching nearly 30% in some samples. The quantitative detection of *Prevotella* spp., both by qPCR and 454 pyrosequencing, was highly accurate for BV (ROC AUC 0.94 and 0.97, respectively).

In our study, BVAB1, BVAB2 and BVAB3 were highly specific for BV. However, of these three species, only BVAB2 was highly prevalent (90% using PCR) in the BV patients, thus ensuring acceptable diagnostic accuracy for BV detection, while BVAB1 and BVAB3 were detected in a minority of the BV patients (34% and 27%, respectively, using PCR). These results are in concordance with previous studies by Fredricks et al. [18], [19].

According to our results, *Megasphaera* type 1 was also highly prevalent and abundant in the BV patients, and its quantitative detection possessed excellent diagnostic accuracy (ROC AUC 0.96 and 0.93 for qPCR and 454 pyrosequencing, respectively). However, *Megasphaera* type 2 was only detected in a minority of the BV patients (10%), thus confirming no discriminatory ability for BV diagnosis. These findings are in agreement with previous studies [18], [19], [25].

The two fusobacteria *S. sanguinegens* and *L. amnionii* were also significantly more prevalent and abundant in the BV patients versus healthy women. The detection of diagnostic levels of *S. sanguinegens* and *L. amnionii* was highly specific for BV prediction. However, as these two bacteria were prevalent in less than 90% of BV patients, the sensitivity of the diagnosis was low.

The remaining BV associated bacteria analyzed, namely, *F. magna*, BVAB-TM7, *M. curtisii* and *M. mulieris*, had poor or no discriminatory abilities for BV. It is noteworthy that we detected *M. curtisii* and *M. mulieris* by PCR in only 43% and 12% of the BV patients, respectively, which is in concordance with previous studies suggesting that *Mobiluncus* species, included in the Nugent score, do not appear to be an appropriate indicator of BV due to the low sensitivity [19], [21], [49].

Lactobacilli are believed to play a crucial role in maintaining the normal vaginal ecosystem by producing lactic acid and other substances that inhibit growth of opportunistic bacteria [50]. With the use of molecular-based techniques, it has been found that the normal vaginal flora is typically dominated by a limited number of *Lactobacillus* species, such as *L. iners, L. crispatus, L. jensenii* and *L. gasseri* [21], [22], [23], [51], [52], [53], [54]. *L. crispatus* and *L. iners* appear to be substantially prevailing over the other *Lactobacillus* species, which was confirmed by our 454 pyrosequencing results. *L. crispatus* has been reported to promote the stability of the normal vaginal microbiota [55]. This bacterium is depleted in BV patients [18], [44], and in a recent study *L. crispatus* was the only species negatively associated with all four Amsel criteria [23]. Our data support these observations; however, since this bacterium is not universally present in all healthy women and is present in many BV patients, it does not appear to be an ideal negative predictor of BV.

*L. iners* is consistently found in large quantities in healthy women, BV patients and women recovering from BV [44], [46], [55], [56], [57]. In an analysis of associations of vaginal bacteria with clinical features, *L. iners* was not associated with any of the Amsel criteria or with the absence of clinical BV characteristics [23]. In our study, *L. iners* was widely present in high loads in both healthy women and BV patients, and no significant differences between these groups in the presence of *L. iners* or in *L. iners* DNA loads were found. However, the relative abundance of the bacterium was significantly lower in the BV patients as compared to the healthy subjects (P = 0.001). Furthermore, our 454 pyrosequencing data showed that *L. iners* was the most abundant species (accounting on average for nearly half of all sequences) in the intermediate cases, which may represent a transitional step between normal vaginal microbiota and BV.

In our study, the depletion of *Lactobacillus* species (relative abundance ≤47%) was shown to be a highly accurate BV predictor. With this criterion, we correctly identified all 73 BV cases (100% sensitivity), while in five of the 79 samples obtained from the healthy women, *Lactobacillus* spp. was also depleted (94% specificity). However, since the normal microbiota can also consist of non-*Lactobacillus* species, such as *Bifidobacterium* [58], and since lactobacilli can be depleted also in other clinical conditions, such as aerobic vaginitis [35], diagnosis of BV cannot solely rely on this approach. When we combined the depletion of *Lactobacillus* spp. with the presence of either *G. vaginalis* or *A. vaginae* at diagnostic levels, the sensitivity and specificity of BV prediction were 97% and 96%, respectively, for qPCR thresholds, and 100% and 95%, respectively, for 454 pyrosequencing thresholds. In one of the five samples from the healthy subjects with *Lactobacillus* depletion, *Bifidobacterium bifidum* predominated, and none of the bacteria known to be implicated in BV was detected. In the remaining four discrepant samples from healthy women, *G. vaginalis* was highly abundant (56–83% of all sequences), with *A. vaginae* also detected in one sample at a proportion of 20%, while *Lactobacillus* species were only present at proportions of 0–26%. In these four samples, all of the Amsel criteria were negative, and in three of the samples, where lactobacilli constituted only 0–3% of all sequences, wet mount microflora was recorded as short lactobacilli. It is known that some small bacterial morphotypes, such as *Gardnerella*, may vary in size and form, from round to elongated, and sometimes it is difficult to distinguish them from *Lactobacillus* morphotypes. This emphasizes the need for objective approaches, capable of accurate quantitative assessment of the representation of normal and BV microflora in vaginal fluid. In our study, relative proportions of bacteria in patient samples were assessed using 454 pyrosequencing. Quantitative PCR, which is more affordable and available than 454 pyrosequencing, can also be used for this purpose. However, with both methods a totally accurate conversion into bacterial counts is difficult, because 16S rDNA is most often used as PCR target and the number of rRNA operons per organism may vary significantly between species. For increased specificity of the BV diagnosis, these 16S rDNA-based approaches could possibly also be beneficially combined with, e.g., identification of sialidase producing *G. vaginalis* strains [59], [60].

Recently, several approaches for molecular diagnosis of BV have been described. Menard et al. [31] found that molecular quantification of *A. vaginae* and *G. vaginalis* had excellent sensitivity (96%) and specificity (99%) when compared with the Nugent scores. A BV PCR assay, developed by Cartwright et al. [37], for detection of *A. vaginae*, BVAB2 and *Megasphaera* type 1 demonstrated a sensitivity of 97% and specificity of 92%, with 5.3% of the samples classified as indeterminate for BV. Besides objectivity and good diagnostic performance, molecular techniques make it possible to more objectively use self-collected samples. In a recent study, the validity and reliability of self-collected swabs for molecular diagnosis of BV was also confirmed [61].

### Conclusions

The results of our study support previous molecular studies showing that BV is associated with substantial compositional changes in the vaginal microbiota. The BV associated bacteria *G. vaginalis*, *A. vaginae*, *Eggerthella*, *Prevotella*, BVAB2 and *Megasphaera* type 1 detected at diagnostic levels, expressed as relative abundances or absolute bacterial genome/gene counts (16S rDNA genome/gene copy numbers), were highly predictable for BV, with the best diagnostic accuracy shown for *A. vaginae*. The depletion of *Lactobacillus* species combined with the presence of either *G. vaginalis* or *A. vaginae* at diagnostic levels was a highly accurate BV predictor. Measurements of abundance of normal vaginal microbiota and BV microflora relative to total bacteria in vaginal fluid may provide more accurate tools for BV diagnosis, including test-of-cure, than qualitative detection or absolute counts of BV related microorganisms. Nevertheless, although the present and previous studies represent important steps toward molecular diagnosis of BV, the generalizability of the results to other populations (patient groups and geographic settings) needs to be established before application of these approaches in clinical practice can be considered. Furthermore, additional knowledge regarding the fluctuations of the vaginal microbiota due to factors such as menstrual cycle, concomitant infections, stress, contraception and vaginal cleansing habits would be valuable.

## Supporting Information

Table S1 **Primers and probes used in quantitative bacterial species/genus-specific real-time PCR assays.**
(XLS)

Table S2 **Detection rates and relative abundances of vaginal bacterial species/genera by 454 pyrosequencing in healthy control women (n = 79), intermediate cases (n = 11) and BV patients (n = 73).**
(XLS)

Table S3 **Detection rates and concentrations of vaginal bacterial species/genera by bacterium-specific real-time PCR in healthy control women (n = 79), intermediate cases (n = 11) and BV patients (n = 73).**
(XLS)

Table S4 **Agreement between the presence of individual bacterial species/genus determined by 454 pyrosequencing and real-time PCR in vaginal samples.**
(XLS)

Table S5 **Diagnostic characteristics of detecting vaginal bacteria for BV prediction.**
(XLS)

Table S6 **Diagnostic characteristics of detecting combinations of** ***Gardnerella vaginalis*** **,** ***Atopobium vaginae*** **and** ***Lactobacillus*** **for BV prediction.**
(XLS)
